# The principles of natural climate solutions

**DOI:** 10.1038/s41467-023-44425-2

**Published:** 2024-01-23

**Authors:** Peter Woods Ellis, Aaron Marr Page, Stephen Wood, Joseph Fargione, Yuta J. Masuda, Vanessa Carrasco Denney, Campbell Moore, Timm Kroeger, Bronson Griscom, Jonathan Sanderman, Tyson Atleo, Rane Cortez, Sara Leavitt, Susan C. Cook-Patton

**Affiliations:** 1 The Nature Conservancy, Arlington, VA USA; 2 Forum Nobis, Iowa City, IA USA; 3 Paul G. Allen Family Foundation, Seattle, WA USA; 4 Conservation International, Arlington, VA USA; 5 Woodwell Climate Research Center, Falmouth, MA USA; 6 Nature United, Victoria, BC Canada

**Keywords:** Climate-change mitigation, Conservation biology

## Abstract

Natural climate solutions can mitigate climate change in the near-term, during a climate-critical window. Yet, persistent misunderstandings about what constitutes a natural climate solution generate unnecessary confusion and controversy, thereby delaying critical mitigation action. Based on a review of scientific literature and best practices, we distill five foundational principles of natural climate solutions (nature-based, sustainable, climate-additional, measurable, and equitable) and fifteen operational principles for practical implementation. By adhering to these principles, practitioners can activate effective and durable natural climate solutions, enabling the rapid and wide-scale adoption necessary to meaningfully contribute to climate change mitigation.

## Abstract (web summary)

Natural climate solutions can mitigate climate change but misunderstandings about what constitutes a natural climate solution generate unnecessary confusion and controversy. This Perspective distills five foundational principles of natural climate solutions and fifteen operational principles for practical implementation.

## Introduction

While the general mechanisms by which plants affect climate have been understood for over a century^1–3^, in 2017 scientists and conservation practitioners framed the holistic concept of ‘natural climate solutions’ (NCS) to adapt existing knowledge and experience to climate action. As originally defined, NCS are deliberate human actions (NCS pathways) that protect, restore, and improve management of forests, wetlands, grasslands, oceans, and agricultural lands to mitigate climate change^4^. NCS were also defined as having no net negative impact on food and fiber supply and no net harm to biodiversity, while ensuring actions are implemented in socially and culturally responsible ways^4,5^.

In the past six years, interest in NCS has increased dramatically. The conversation has tripled in size, from < 2% to > 6% of climate-related social media traffic (see Supplementary Methods), and funding commitments have doubled^6^. However, this pace must accelerate exponentially if we are to succeed^7^. The Intergovernmental Panel on Climate Change (IPCC) emphasizes that the rapid deployment of NCS (which the IPCC calls Agriculture, Forestry and Other Land Use [AFOLU] mitigation measures) is essential to reach net zero emissions and avoid catastrophic warming, but if deployed carefully and appropriately it can deliver a third of the climate mitigation needed by 2030. However, investments will require > $400 billion per year^8^, which is over nine times the amount being spent today^9^.

**Fig. 1:**
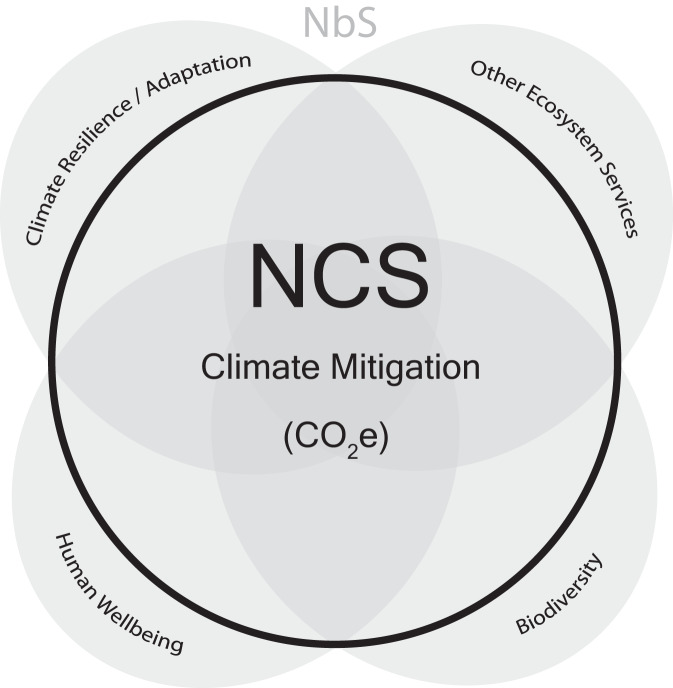
Overlap of natural climate and nature-based solutions. Conceptual diagram showing the overlap between nature-based solutions (NbS) and natural climate solutions (NCS). While NCS focus on have a single outcome (CO_2_ equivalents; CO_2_e), NbS can be defined by multiple outcomes with multiple metrics.

Accompanying this uptick in interest has been a concomitant rise in confusion and controversy. In some instances, the excitement around NCS has led to well-intentioned but hastily and poorly designed tree planting programs, which have rightly catalyzed heated dialog around considerations for implementing NCS programs^10^. In other instances, NCS have been dismissed as greenwashing because they are “vulnerable to exploitation by companies that want to appear at the vanguard of climate action^11^.” A similar misperception portrays NCS as predominantly carbon offsetting mechanisms promoted by energy intensive industries^12^. Another confusion arises from the overlap between NCS and carbon dioxide removal (CDR); some NCS (for example reforestation) do indeed remove carbon dioxide (CO_2_) from the atmosphere, but others (avoided peatland conversion) avoid CO_2_ or other greenhouse gas emissions. Furthermore, NCS are often conflated with other terms such as nature-based solutions (NbS), nature-based climate solutions (NbCS) and AFOLU. Compared with NCS, NbS refer to a much broader set of actions that address a range of societal challenges beyond only climate mitigation (Fig. 1)^13–15^; NbCS are nearly identical to NCS but include some additional activities in engineered ecosystems (for example, macroalgae farming^16^) that have been moved further from their natural state (Principle 1.2 below)^17,18^. NCS are synonymous with AFOLU mitigation measures as defined by the IPCC^8^.

Perhaps another source of confusion is driven by the fact that the NCS concept builds upon a long history of conservation science and practice but focuses the framework on measurable climate change mitigation. For example, the United Nations’ reducing emissions from deforestation and forest degradation in developing countries program (REDD+), payment for ecosystem services, community-based conservation programs, national government conservation incentive programs, and other well-established and studied interventions or programs all presumably fall under the umbrella of NCS and provide a rich and diverse body of evidence on advances and challenges in NCS implementation. The original NCS study^4^ was powerful because it explicitly estimated the climate change mitigation potential of known and long-studied conservation interventions, providing insight on whether, which, and to what extent natural solutions could feasibly advance global climate change mitigation goals. One challenge that emerged from the analysis was that there is no guarantee that past interventions and programs were truly climate additional even though they plausibly fit within the NCS umbrella. Meanwhile, several studies have been conducted that provide insights on the determinants of successful NCS projects which can inform future action^19–22^.

Finally, significant confusion arises when untested NCS interventions are promoted widely without a robust scientific basis. However, recent publications have provided a mechanism for differentiating well-tested ‘ready-to implement’ NCS pathways from emerging or nascent pathways that require further research^17,23^.

While confusion and controversy are to be expected in a rapidly growing field, without a clear framework for productive conversation and reliable action, there is a concern that momentum for NCS implementation will stall. Avoiding climate catastrophe requires immediate NCS action^24,25^, but to be effective, NCS must be implemented equitably and sustainably. When initiatives claim to implement NCS but fail to achieve sustainable, equitable climate mitigation, they divert resources away from legitimate climate solutions and undermine public support for true NCS.

Many have commented on the risks of poorly-informed NCS action^18,19,21,26–28^, but few have offered a clear path forward for real, fair and well-informed NCS action. If the NCS movement is to scale rapidly, the evolving NCS conversation needs normative criteria to help practitioners, policymakers, researchers, and the public evaluate whether NCS options are tangible, viable, and appropriate.

**Fig. 2:**
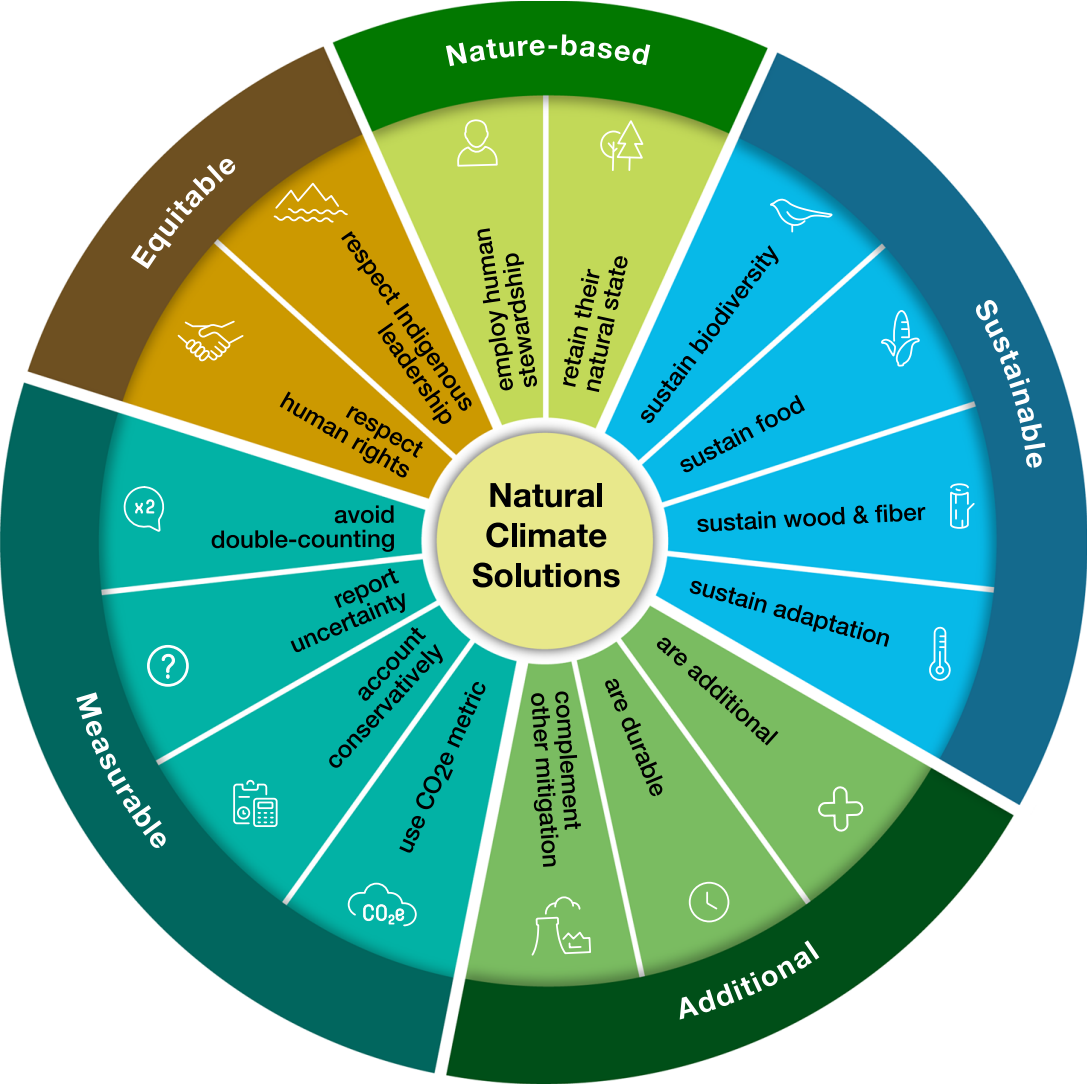
The wheel of natural climate solutions. Foundational principles are shown along the outer edge of the wheel, while operational principles are the ‘spokes’ inside the wheel. See Box 1 and main text for the full definition of each principle.

In this Perspective, we outline these normative criteria as a set of NCS principles (Box 1 and Fig. 2) that can be used to identify NCS actions worthy of support. These principles address problems that contribute to unproductive confusion and controversy around climate change mitigation. NCS foundational principles are criteria (nature-based, sustainable, climate-additional, measurable, and equitable) that provide a working definition of NCS based on the existing literature. This working definition enables more effective and productive NCS action by clarifying the boundaries of the NCS conversation. NCS operational principles guide NCS implementation by specifying how to apply NCS foundational principles to real-world action. These fifteen operational principles provide guidance to policymakers and practitioners so that risks can be navigated intelligently without impeding action. Taken together, the NCS principles orient NCS activities to the appropriate scope (Principle 1), ensure positive climate benefits (Principle 4) and avoid negative impacts (Principles 2, 4, and 5). While we hope the normative nature of these principles facilitates implementation by mitigating against confusion and controversy, we acknowledge that, as with all normative criteria, they are idealized, and the real-world complexities of conservation action will continue to expose new uncertainties that should allow the concept to evolve through active and adaptive action. Over time, it may be necessary to add or adjust principles.

Box 1 The principles of natural climate solutions Foundational principles define natural climate solutions (NCS). operational principles (for example, Principle 1.1, 2.1) guide NCS implementation by specifying how to operationalize foundational principles.

**Foundational Principle 1: NCS are Nature-based.**

Principle 1.1: NCS result from the human stewardship of ecosystems.

Principle 1.2: NCS do not move ecosystems further from their natural state.

**Foundational Principle 2: NCS are Sustainable.**

Principle 2.1: NCS sustain biodiversity.

Principle 2.2: NCS sustain food production.

Principle 2.3: NCS sustain fiber and wood production.

Principle 2.4: NCS sustain climate adaptation services.

**Foundational Principle 3: NCS are Climate-additional.**

Principle 3.1: NCS provide additional climate mitigation that would not happen without human intervention.

Principle 3.2: NCS provide durable mitigation.

Principle 3.3: NCS are not used to compensate for readily abatable emissions.

**Foundational Principle 4: NCS are Measurable.**

Principle 4.1: NCS are quantified in terms of cumulative effects on radiative forcing.

Principle 4.2: NCS accounting is conservative.

Principle 4.3: NCS with uncertainty ranges greater than the estimated climate mitigation should be flagged as emerging.

Principle 4.4: NCS accounting avoids double-counting.

**Foundational Principle 5: NCS are Equitable.**

Principle 5.1: NCS respect human rights.

Principle 5.2: NCS respect Indigenous self-determination.

## Foundational principle 1: NCS are nature-based

### Principle 1.1: NCS result from the human stewardship of ecosystems

NCS involve active management decisions that affect human stewardship of ecosystems and result in net climate mitigation. An ecosystem is broadly defined to include all the living organisms, including humans, and their interrelationships within a physical environment. Notably, this definition includes both natural and working lands. For example, agroforestry can lead to carbon sequestration in soil and woody plants^29^ and occurs within an ecosystem involving farmers, crops, soil, and the soil microbial community. Principle 1.1 acknowledges that, despite common misconceptions of nature as something devoid of humans, humans have shaped natural lands and waters for millennia^30^.

Changes in human stewardship of ecosystems can be triggered locally by supply-side decisions (for example, a rancher adopting silvopastoral practices) or demand-side decisions (for example, an urban resident deciding to stop eating beef). While we acknowledge the influence of demand-side climate solutions such as diet change, shifts to longer-lived wood products, reduced food waste, or biofuel usage, it is beyond the scope of this paper to trace these supply and demand-side interactions for each NCS Pathway. IPCC refers to the interacting aggregate of supply and demand-side actions as AFOLU mitigation measures^8^. Conservation International groups AFOLU mitigation measures into people-centered (rather than ecosystem centered) NCS Action Tracks^31^.

### Principle 1.2: NCS do not move ecosystems further from their natural state

Being nature-based also means that NCS stewardship does not move ecosystems further away from their natural state than they are already. Elements of structure, composition, and function of the unmodified, naturally occurring system must be considered, as well as the current land use. For example, while tree planting may be perceived as a positive activity, replacing a natural forest by planting a plantation of non-native tree species, even if faster-growing, would not be considered an NCS, because the natural structure and function of the forest would be diminished as a result^32^. Similarly, artificial fertilization of ocean water to stimulate algal bloom^33^, ocean alkalinization^34^, and kelp/seaweed afforestation^35^ would not be considered NCS because human intervention moves the ecosystem farther from its unmodified state.

In many cases, advocates and practitioners will need a nuanced and dynamic understanding of an ecosystem’s natural state to decide whether an intervention adheres to Principle 1.2. The structure and composition of many ecosystems are transitioning as climate changes, and some need human assistance to become more climate resilient. Therefore, careful assessment of actions is often needed to determine what movement away from, or towards, a natural, climate-resilient state means in practice. For example, if there is clear evidence that a forest ecosystem is transitioning into a grassland ecosystem in response to climate change, then restoration NCS should be aligned with these dynamics.

## Foundational principle 2: NCS are sustainable

### Principle 2.1: NCS sustain biodiversity

Activities that transition an ecosystem further away from its unmodified state would also fail to qualify as NCS because NCS should sustain biodiversity. NCS can have neutral near-term local impacts, but must avoid reductions in alpha, beta, and/or gamma diversity as is described in the Convention on Biological Diversity^4,36^. For example, turning agricultural residue into soil biochar stores carbon in the soil without harming biodiversity^37^. In contrast, adding trees to native grasslands may increase carbon sequestration at the expense of native grassland diversity and thus would not qualify as an NCS^38^.

### Principle 2.2: NCS sustain food production

NCS should sustain food production. Climate solutions will not be durable unless food security can be provided alongside farmer and fisher livelihoods^39^. Maintaining food security in the face of a growing human population and changing diets is a complex challenge. Extensive reforestation on cultivated lands and constraints on agricultural inputs (for example, fertilizer use) can negatively impact food security^40^. However, various combinations of maintaining existing cropland^4^, improving fertilizer management in ways that reduce the cost of crop production^41^, implementing silvopasture to increase livestock productivity in existing pastoral systems^42^, limiting bioenergy production and associated land demand^43,44^, and adopting more climate-friendly diets enable NCS implementation while increasing food security^45^.

### Principle 2.3: NCS sustain fiber and wood production

NCS should sustain fiber and wood production as climate solutions will not be durable unless rising wood product demand can be met while maintaining forest-based livelihoods^46^. Wood-based materials are particularly important to the success of sustainable climate mitigation because, when well-sourced through selective logging and existing forest plantations, they have much smaller carbon footprints than building alternatives such as steel and concrete. However, unsustainable timber exploitation that involves deforestation or that harvests forests above a scientifically defined level of sustained yield^47^ would undermine this principle and would therefore not be considered an NCS.

### Principle 2.4: NCS sustain climate adaptation services

Finally, while NCS are focused on climate mitigation outcomes, at a minimum they sustain climate adaptation services through the ecosystems in which they are implemented. A rich literature identifies the multitude of ecosystem and climate adaptation services that NCS can provide, such as attenuation of floods, soil erosion, landslides, storm surges, and the resulting human benefits^48–53^. However, existing ecosystems often already provide adaptation services. Any implementation of NCS should at sustain existing levels of adaptation services to ensure adaptation is co-produced alongside mitigation.

Climate change is a significant threat to people, biodiversity, and other ecosystem services, and NCS offer real near-term climate mitigation that alleviates this threat. Thus, over longer time scales, NCS deliver positive benefits to ecosystems and people, either directly (by providing net positive local effects) or indirectly (by sustaining the climate stability upon which these benefits rely). A comprehensive approach to climate change must consider both mitigation and adaptation, and projects that provide both, as many NCS projects do, are particularly valuable in this regard.

Note that Principles 2.1–2.4 are sensitive to scale. For example, a 100-hectare cropland reforestation NCS project may fail to sustain food production at the project scale, but a state-wide reforestation program may not, if any lost cropland is either marginal or accommodated through intensification.

While NCS does not by definition require additional non-climate benefits, on-the-ground NCS projects are frequently implemented to achieve multiple co-benefits. Indeed, the promise of NCS co-benefits is often a primary motivating factor for decision-makers, especially when juggling multiple conservation and climate priorities alongside other sustainable development goals. Therefore, it is important for researchers to continue compiling evidence for decision-makers to understand whether and to what extent NCS provide co-benefits (or involve tradeoffs).

## Foundational principle 3: NCS are climate-additional

### Principle 3.1: NCS provide additional climate mitigation that would not happen without human intervention

NCS provide additional climate mitigation that would not happen without human intervention. Additionality is traditionally assessed in reference to a ‘business as usual’ baseline scenario^54^. For example, establishing a forest reserve in a remote landscape with high carbon stocks would not count as NCS unless that landscape was threatened by human disturbance. If that landscape is not threatened, then reserve status does not alter the fate of the carbon stocks stored within it. Similarly, in locations experiencing land abandonment and natural recovery of native ecosystems, it would not be appropriate to count pre-existing recovery as NCS, because that recovery is part of the baseline condition and not specifically associated with human intervention. However, cleared land that is expected to remain cleared (for example, as a pasture), and where a deliberate choice is made to instead allow natural recovery, would count as NCS because human intervention changes the trajectory of the land use. Note that this principle applies to both avoided emissions (for example, avoided forest conversion) and CDR (for example, reforestation) NCS.

**Fig. 3:**
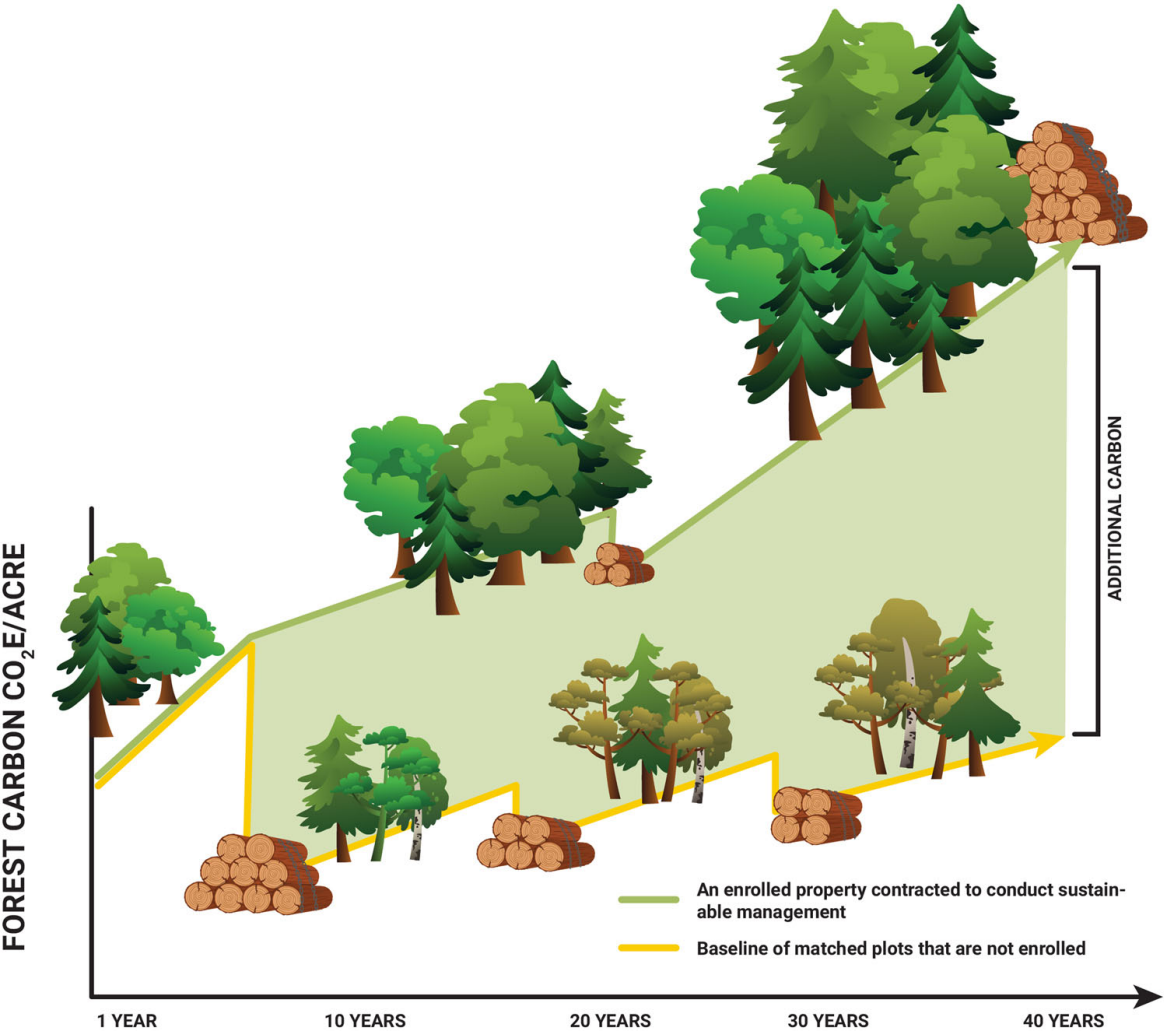
Schematic representation of additionality calculations. Figure shows how additionality (Principle 3.1) is calculated for improved forest management projects within the Family Forest Carbon Program (FFCP)^108,109^. Increasing stocks over time represent long term increases in timber and carbon yields predicted under FFCP practices, ensuring sustainability (Principle 2.3). Periodic harvests show that FFCP is committed to maintaining working forests (Principle 1.1). Adapted from the Dynamic Baselines infographic with permission from The Nature Conservancy.

Methods for demonstrating additionality are varied, and each approach has strengths and weaknesses. The scientific community that supports NCS action should commit to continuous improvement focused on methodological transparency, accuracy, and efficiency. For example, traditional baseline approaches to forest NCS measurement are being updated with dynamic global monitoring systems that can compare projects to matched control sites and track benefits as they accrue using coordinated networks of field measurements and improved remote-sensing technologies (see Fig. 3)^55^.

### Principle 3.2: NCS provide durable mitigation

NCS must also provide durable mitigation, meaning that additional climate benefits persist over time. Durability is defined in terms of different greenhouse gas (GHG) residence times in different pools (for example, soil or aboveground biomass) at different scales (for example, national or global), but should avoid binary classifications (that is, permanent versus impermanent)^56,57^. The key consideration is whether the NCS activity will provide mitigation for a long enough period to deliver measurable, additional, net positive climate benefits^58^. For example, peatland rewetting initiatives might not qualify as NCS if the restored peatland is not maintained long enough to counterbalance the adverse warming effect of the methane emissions pulse from rewetting^59^.

Many ecosystems stewarded through NCS have proven extremely durable. For example, Australia’s Daintree Rainforest has effectively stored carbon for 135 million years, and is currently protected as a national park^60^. Other ecosystems are less durable, either due to human or environmental threats, such as conversion for urban development or increased wildfire risk due to a warming climate^61,62^. To adequately account for this variation, NCS should be considered in the context of both the imperative to act now, and the imperative to simultaneously build robust systems to ensure durability over time. Enabling conditions are needed to ensure NCS durability at adequate spatial and temporal scales; for example, a global NCS monitoring system is needed to detect and quantify reversals, and long-term insurance and financial systems are needed to ‘pay back’ the atmosphere in the event of reversals. Carbon market standards are in the process of developing these systems, but more research is needed to calibrate these systems with quantifiable risks of reversal^58^. As with principle 3.1, durability considerations are equally important to CDR and avoided emissions NCS.

### Principle 3.3: NCS are not used to compensate for readily abatable emissions

It is imperative that NCS not be used to compensate for readily abatable emissions. Non-NCS strategies that reduce emissions from fossil fuels will need to deliver the majority of the mitigation required to meet Paris Agreement goals^60,61^. NCS must proceed in parallel to these strategies. In most cases, this is unproblematic. Governments and other climate mitigation actors may choose to invest in or focus on NCS for many legitimate reasons, including cost efficiency and the multiple layers of co-benefits offered by some NCS. But when ecosystem-based mitigation is used to offset readily abatable emissions in another sector it provides no real climate benefit.

Application of principle 3.3 requires nuance and consideration of the different venues in which climate progress is pursued (for example, public sector commitments or voluntary carbon markets (VCM)). Different actors will have different views on complex questions of what emissions are ‘readily abatable’, but establishing clear best practice across different sectors and contexts is necessary to maintain societal buy-in. This principle can accommodate such differences while also serving as a foundation to resist non-credible claims. At a minimum, parties seeking to use NCS carbon credits to compensate for unabated emissions should be able to have a record of and target for abatement, articulate why the emissions to be compensated are not currently abatable, and locate the credits on a path to reach a legitimate mitigation goal under the Paris Agreement or applicable private sector standards. Notably, many efforts are underway that can help corporations and other entities align their emissions reduction goals with a science-based path to limit global warming and identify ‘residual’ emissions that would not have been abated even under ambitious decarbonization scenarios^62–64^. These demand-side integrity programs require further development to ensure true ‘atmospheric additionality’ of mitigation but are both necessary and sufficient to allow the purchase and trade of NCS credits in a way that will accelerate rather than delay global climate progress^65^.

It is possible that NCS carbon market projects will be developed without knowing—or being able to control—how generated credits will later be used by purchasers. This means that we need to push for systems that enable greater transparency (for example, the demand-side integrity programs mentioned above) so that the stakeholders who generate the credits can better choose to whom they sell credits. Principle 3.3 speaks directly to purchasers as key participants in the overall solution.

## Foundational principle 4: NCS are measurable

There are multiple potential NCS actions that can occur in a given landscape and quantifying the overall magnitude of opportunity can help to focus efforts on the actions that can offer the largest mitigation returns. However, appropriate accounting is required to ensure that NCS potential is consistently and clearly quantified.

### Principle 4.1: NCS are quantified in terms of cumulative effects on radiative forcing

NCS mitigation is quantified in terms of cumulative effects on radiative forcing due to changes in stewardship of ecosystems. For consistent comparison, radiative forcing (W m^−2^) is converted to CO_2_ equivalents (CO_2_e) and, for GHGs, considered as a function of GHG flux into or out of the atmosphere^3^. Avoided emissions and removals NCS are treated equally, as their effect on the quantity of GHGs in the atmosphere is the same. GHGs to be considered include CO_2_, methane (CH_4_), and nitrous oxide (N_2_O) fluxes. Ideally, NCS mitigation estimates also incorporate biophysical factors that affect top-of-atmosphere radiative forcing, including black carbon deposited from particulate matter, changes in albedo resulting from changes in land cover, and changes in water vapor^66^. These first two non-GHG factors have the potential to significantly alter the net climate impact of NCS and thus shape where, when, and how NCS implementation occurs. Although these factors have been difficult to directly quantify, there is opportunity for them to be included as the science improves. Failing to consider all climate forcing agents could lead to adoption of NCS that have little climate benefit. For example, tree planting in dry forest areas may appear to be net climate positive when quantifying carbon, but actually might be net negative after considering changes in albedo^67^.

To better facilitate comparisons, it is possible to translate non-CO_2_ climate pollutants into CO_2_e using established Global Warming Potentials (GWP)^8^(see section 7.6 of Ref. ^8^). How to make these comparisons depends on whether one is accounting for near-term or long-term climate impacts. To appropriately consider both near-term and long-term climate impacts, we recommend accounting for short-lived and long-lived climate pollutants using different GWP conversions. For example, following IPCC guidance, GWP_100_ is appropriate to calculate the climate impact of NCS over the long term and for long-lived climate pollutants such as CO_2_ and N_2_O^68^. However, conversion equations such as GWP* are better suited to account for the climate impact of short-lived climate pollutants, such as CH_4_ and black carbon^69^. In this way, targets and monitoring systems for long and short-lived climate pollutants can be accounted for separately, thereby enabling incentives to be developed appropriate to the timing of their atmospheric impacts^56^.

### Principle 4.2: NCS accounting is conservative

NCS accounting adheres to the convention of conservativeness^70^, whereby data^56^ are considered only when sufficient evidence exists to support their inclusion. Buma et al.^17^. have recently assessed the strength of the science underlying different NCS actions and show how some NCS pathways (for example, avoided forest conversion) have well-constrained estimates of mitigation, but others (such as avoided benthic disturbance) include unresolved or incomplete accounting. Sufficient evidence means that additional NCS mitigation potential estimates are significantly different from zero with medium or greater confidence according to the IPCC^68^. New NCS pathways and activities can be added as the science evolves and estimates improve. For example, data on rates of urban tree cover loss in Canada enabled estimates of the mitigation potential of maintenance of urban tree cover in that country^71^, allowing this pathway to be incorporated into NCS mitigation assessments^72^. Some pathways may also delineate new activities as more refined data becomes available. For example, global estimates of climate-smart forestry NCS (originally termed natural forest management) were initially calculated in aggregate^4^, but more recent research has begun to parse this into specific activities such as reduced-impact logging for climate mitigation^73^ and liana removal^74^.

### Principle 4.3: NCS with uncertainty ranges greater than the estimated climate mitigation should be flagged as emerging

Determining whether a candidate NCS pathway will provide significant mitigation requires estimating the uncertainty of the quantified NCS mitigation. NCS with uncertainty ranges greater than the estimated climate mitigation should be flagged as emerging. Robust and complete assessments of uncertainty can and should affect decision-making. For example, Griscom et al.^4^. identify reforestation as the single largest NCS pathway based on biophysical potential (10.1 PgCO_2_ yr^−1^), nearly equal to all other NCS mitigation potentials combined. However, the 95% confidence interval is 74% of the mean estimate (7.5 PgCO_2_ yr^−1^). If reforestation offered only 2.6 PgCO_2_ yr^−1^, it would dramatically alter the implications for prioritizing reforestation globally. Quantifying and reporting NCS uncertainty at the appropriate scale is important to ensure that incentives are focused on actionable NCS, and research is focused on emerging NCS^17,23^.

### Principle 4.4: NCS accounting avoids double-counting

NCS accounting should make all attempts to avoid double-counting. For example, when estimating NCS opportunity, a given pasture could be reforested or could have improved grazing management activities, but not both. In contrast, some NCS pathways can be deployed in parallel, such as agroforestry practices and biochar application on the same agricultural land. Careful consideration of which pathways can and cannot be applied on the same area at the same time is necessary to avoid overstating (see Principle 4.2) or understating the potential of NCS on a given landscape. The original NCS publication established a series of hierarchical rules to eliminate double counting, described in its supporting information appendix (see Supplementary Note 1)^4^. Similarly, when generating carbon credits or reporting toward country-level or other jurisdictional targets, NCS projects should follow accounting best practices to ensure that the mitigation they provide is not double counted within emission inventories or carbon market schemes. To avoid double counting, it is helpful to use the NCS framework to categorize activities into biomes, conservation actions, and pathways (see Supplementary Note 1). Note that conservation pathways related to protection measure avoided emissions, while those related to restoration measure additional sequestration. In contrast, improved management pathways can be a mix of both increased sequestration and avoided emissions. For example, agroforestry increases C sequestration, but biochar and no tillage (contrary to some misconceptions)^75^ avoid emissions via decomposition of vegetation or avoided oxidation of soil carbon.

## Foundational principle 5: NCS are equitable

The original NCS publication emphasized that “work also remains to refine methods for implementing NCS pathways in socially and culturally responsible ways”^4^_._ This statement was a call for additional research and action, but also a recognition that NCS must be equitable. The need for a social equity lens is driven by historical and ongoing injustices associated with management of natural resources^76,77^. Vulnerable populations such as Indigenous Peoples, local communities, farmers, forest managers, coastal communities, conservationists, women, and other marginalized groups are often the least responsible for historic emissions, bear the greatest costs and impacts of climate change^78^, and yet are often the most active and effective NCS stewards^79–83^.

Despite best practices in conservation around rigorous review procedures and community consultation, recent scholarship has identified deeper equity issues in the field that persist and that have been explored through a number of frameworks such as the capabilities approach^84^, the ‘Just Sustainabilities’ concept^85^, human rights^86^, and Indigenous Peoples’ rights^87^. This critical lens reveals a new set of challenges: while equality of participation and material outcomes remains important, true equity may require reimagining underlying root concepts to consider previously marginalized and excluded interests and experiences. Environmentalism, conservation, sustainability, and similar root concepts must be understood as culturally embedded and linked to particular social identities and political choices, rather than as abstract, inherent, and universal.

More concretely, two studies present an analytical framework that recognizes multiple dimensions of equity, including procedural (involvement and inclusiveness of all rightsholders and interested parties), distributive (fair allocation of costs, benefits, burdens, and rights), recognitional (respect for knowledge systems, values, social norms, and rights of all rightsholders and interested parties), and contextual (attention to power dynamics and the social conditions that affect ability to advocate for equity on the other dimensions)^88,89^. Many social equity safeguard frameworks for use with NCS have already emerged and are being piloted^90–92^. While NCS can work toward social equity in myriad ways, the following principles can help to deliver socially and culturally responsible NCS implementation and meet the basic commitment to equity.

### Principle 5.1: NCS respect human rights

NCS should respect human rights. This means that NCS activities comply with national laws and international human rights law, as reflected in the International Bill of Human Rights^93,94^, the International Labor Organization Fundamental Principles and Rights at Work^95^, the United Nations Declaration on the Rights of Indigenous Peoples (UNDRIP)^87^, and other key conventions and sources. For example, the use of financial and legal resources to acquire land used customarily by subsistence farmers who lack the resources to acquire legal title might be allowed by a national legal system but would be considered a ‘land grab’ linked to violation of internationally recognized human rights, and thus would not be acceptable to advance NCS^96^.

NCS projects should be able to demonstrate respect for human rights. This usually means a policy foundation and a ‘due diligence’ or assessment practice that helps an NCS project identify potential human rights impacts. Drawing from existing practice under the UN Guiding Principles on Business and Human Rights, NCS proponents should strive for human rights policies^97^ that acknowledge human rights frameworks, identify priority risk areas and corresponding safeguards, and explain how people who experience a rights violation related to NCS implementation can bring it to the attention of jurisdictional authorities and NCS project managers. NCS proponents should strive for due diligence practices^98^ that define and continuously assess key indicators of human rights risk and invite people directly affected to co-create any needed mitigation strategies, thus supporting the multiple dimensions of procedural, distributional, and recognitional equity. Further, NCS projects should mainstream gender equity considerations^99^ in all design and implementation processes and should focus due diligence and mitigation efforts on vulnerable groups and identities.

Consistent with Principle 4.2 and the convention of conservativeness, NCS activities should not proceed in the face of allegations or concerns about specific human rights impacts until a due diligence system is in place to demonstrate how the impacts have been considered and addressed in a manner consistent with the multiple dimensions of equity.

### Principle 5.2: NCS respect indigenous self-determination

As a subset of human rights that particularly relates to NCS implementation, NCS should respect indigenous self-determination, including governance, knowledge, and spirituality. As such, NCS projects should aim to enhance local leadership and decision-making for both Indigenous Peoples and local communities generally.

Self-determination is a multi-dimensional collective right, most clearly articulated and protected in the UNDRIP (Art. 3)^87^. It includes enumerated articles recognizing specific indigenous collective rights, including the right to autonomy or self-government in internal or local matters (Art. 4), the right to participate in decision-making (Art. 18), the right to determine and develop priorities and strategies (Art. 23), the right to territories and resources (Art. 32), the right to give or withhold Free Prior and Informed Consent (FPIC) (Arts. 19, 32.2), the right to protect and strengthen histories, languages, oral traditions (Art. 13), cultures (Art. 15), spiritual and religious traditions (Art. 12), distinctive spiritual relationships with lands (Art. 25), traditional knowledge and cultural expressions (Art. 31), and institutional structures and practices (Art. 34)^87^. The ways in which different NCS actions might contribute to or detract from the various social, economic, and political dynamics and processes related to self-determination are deeply context specific^100^. In most cases, respect for self-determination will require promoting indigenous leadership or deep collaboration in decision-making throughout the design, implementation, monitoring, and benefit-sharing of any project or program affecting Indigenous People.

Principle 5.2 requires that all NCS actors respect FPIC rights for Indigenous Peoples, consistent with the UNDRIP. NCS actors should also ensure FPIC for any local community that could be significantly affected. Numerous tools are publicly available to help NCS actors understand and ensure FPIC is carried out^101–105^. The self-determination of local communities can and should be amplified by preserving or increasing local decision-making and control over key priorities and strategies.

It is particularly important that NCS implementation not increase security threats faced by Indigenous People or local communities, nor result in dispossession or increased pressure on communities that use land on a customary but legally insecure basis. NCS can best avoid such outcomes by embedding respect for human rights and self-determination into project design and implementation activities. A failure to demonstrate FPIC or address human rights risks could make an NCS project ineligible to register carbon credits on the compliance or voluntary carbon markets, or under the anticipated Article 6 of the UN Paris Agreement^106^_,_ and therefore could undermine the ability to achieve national climate goals. Strengthened self-determination can activate critical local knowledge and add valuable local experience to the global NCS learning and science community. NCS projects that demonstrate respect, responsibility, and equity will be more resilient, will inspire action rather than controversy, and will better advance the climate solutions that we so urgently need.

## Monitoring and policy considerations

For NCS to effectively contribute to climate change mitigation, it is critical that there exist robust systems for measuring, monitoring, reporting, and verifying (MMRV) net emissions changes as a result of NCS implementation. There are widespread efforts to advance these systems, such as science and technology measurement systems for corporate supply chain inventory accounting methods (for example, Greenhouse Gas Protocol) and efforts to establish best practice MMRV through global initiatives like the Integrity Council for Voluntary Carbon Markets (IC-VCM). These initiatives are a good start and appropriately recognize the need for continual improvement. There remain important accounting uncertainties, principally in scientific best practice for establishing the baseline/counterfactual scenario against which NCS progress is measured, and leakage. Advanced remote sensing, machine learning, and impact evaluation methods from other disciplines offer rich near-term opportunities to establish a new high bar of NCS accounting. The scientific community should strive for consensus in best practices to give markets and policymakers the certainty needed to support NCS implementation at large scales. However, there remain critical uncertainties and gaps in these systems, such as whether outcomes can be accurately quantified at large scales, or how to align accounting across scales without double counting from project to value chain and national inventory. It is not a foregone conclusion that we will be able to adequately achieve the ambition of developing high quality global MMRV systems for NCS, but if we are to succeed in realizing 11 Pg of cost-effective global NCS potential, a diverse and concerted effort to accelerate the development of high-quality global MMRV systems for NCS is needed^4,24^.

In this vein, these NCS principles could be used to inform efforts to achieve high quality NCS in multiple fora. In the VCM, initiatives like the IC-VCM (focused on supply side quality) and the Voluntary Carbon Markets Integrity Initiative (VCMI) (focused on demand side integrity) should align updates of their rules with these principles. Carbon buyers and investors in the VCM should ensure their market activity aligns as well. Similarly, crediting protocols for regulatory or compliance carbon markets should be modified to calculate project credits based on the change in total radiative forcing, characterize uncertainty around mitigation, demonstrate compliance with human rights due diligence practices and indigenous self-determination, and align with global best practice on use of carbon credits for compliance purposes. In short, NCS credits can be used to close the gap between readily abatable emissions and the ambition needed to meet the Paris Agreement. But NCS credits should only be used for residual emissions. This approach will require defining what counts as ‘residual’ in each industry, which will need to be based either on unit abatement cost (preferred but difficult to verify) or technology (suboptimal, but readily verifiable). There currently exist >30 compliance carbon markets ranging in jurisdictional scale from subnational (for example, California’s Compliance Offset Projects) to supra-national (the European Union’s Emissions Trading Scheme)^107^, so the effort required to promote NCS principle adoption by even a sizable share of these schemes will be substantial. Another potentially powerful mechanism would be the incorporation of these principles into a country’s Nationally Determined Contributions (self-defined national climate pledges under the Paris Agreement). This would likely take the form of voluntary individual country commitments unless the Paris Agreement signatories make compliance with the NCS principles mandatory for AFOLU commitments in Nationally Determined Contributions.

## NCS exemplar

The Family Forest Carbon Program (FFCP) is an NCS initiative launched by the American Forest Foundation and The Nature Conservancy in early 2020^108,109^. In an effort to solve the inequitable market access of existing forest carbon projects, the FFCP was specifically designed to deliver measurable, additional climate mitigation (Principles 3 and 4) in managed natural forests (Principle 1) while maintaining a sustainable supply of timber (Principle 2) and equitable carbon revenue for small landholders in the United States (Principle 5). Since its inception, the FFCP team has validated an improved forest management methodology through Verra’s Verified Carbon Standard (VCS)^110^, and enrolled > 400 small landowners in climate-smart forestry practice agreements on nearly 60,000 acres. The FFCP is currently undergoing VCS project validation and initial verification, intending to deliver a first tranche of credits to vetted buyers in early 2024. We believe that FFCP adheres to the spirit and practice of the NCS principles, while continuing to evolve and improve (see Supplementary Note 2).

## Outstanding challenges

While we have attempted to resolve some of the persistent confusion and controversy around NCS through the articulation of foundational and operational principles, many real issues remain where critiques and debates will be fruitful. First, much more work is needed to understand and address the feasibility constraints (inputs, markets, behaviors and attitudes, institutions, policies, and governance) that limit NCS action. Second, many ecosystem stewards view NCS benefits in the context of a broader set of benefits (for example, biodiversity, water, air, soil, human well-being, climate resilience); but more work is needed to quantify where and how NCS action can deliver these co-benefits, and where and how there are real trade-offs. Third, continuous effort is needed to ensure that NCS are indeed additional, especially to the extent that NCS activities contribute credits to carbon markets. Fourth, additionality is notoriously difficult to prove in areas with high carbon stocks and low historic rates of disturbance (such as high forest cover, low deforestation zones), despite real increasing future threats; the degree and timing of risk that these forests face needs to be better quantified to determine the relative additionality of ongoing actions to protect them^111,112^. Fifth, additional research is needed to ensure that NCS mitigation remains durable to future disturbances, especially the droughts and wildfires that are expected to increase with climate change. Sixth, NCS science has, to date, largely focused on measuring NCS opportunity, but to be successful, we need consistent, compatible NCS monitoring systems to accurately quantify impacts and learn adaptively. Seventh, as we expand the scale of NCS action in an adaptive management cycle, we need a rapid global learning network to replicate successes and prevent repeating mistakes. Finally, the NCS community as a whole needs to demonstrate a commitment to equity by creatively and continuously seeking ways to recognize and integrate the leadership and, with their consent, the knowledge and experience of Indigenous Peoples and other NCS stewards.

## Conclusion

The coauthors of this paper believe it is time for NCS action. Debate and discussion are a healthy component of applied science, and NCS is no exception. But the urgency of our climate predicament requires human society to adopt a culture of adaptive management, in which climate solutions (natural and otherwise) can adapt rapidly and transparently, in concert with their widespread adoption. The foundational and operational principles outlined in this paper are intended to help resolve confusion to expedite action while also fostering discussion and learning focused on important outstanding questions. Many fora are emerging for this type of productive action-oriented NCS learning and conversation (for example, naturebase.org and restor.eco). We hope that these principles facilitate urgent, productive, and collective action toward the widespread adoption of robust NCS projects.

### Supplementary information

Supplementary Information
