# Supplementary material for: The principles of natural climate solutions
Source: Nat Commun. 2024 Jan 23;15:547. doi: 10.1038/s41467-023-44425-2 (PMC10805724; doi:10.1038/s41467-023-44425-2)
Supplement: Supplementary file 1 — Supplementary Information [file 41467_2023_44425_MOESM1_ESM.pdf]

Supplementary Methods

To assess the changing relevance of NCS in public conversation, an analysis was conducted that counted NCS mentions compared to broader climate change mentions on Twitter between March 2018 and February 2023.

NCS mentions were triggered by the following boolean string: ("Natural Climate solution" OR "Natural Climate solutions" OR NaturalClimateSolution OR NaturalClimateSolutions OR Nature4Climate OR ((Nature OR Natural) AND Climate AND Solutions) OR ((Reforestation OR (Forest AND (Protect OR Protect OR prevent)) OR (Cropland AND nutrient) OR "Conservation agriculture") AND (solution OR Climatechange OR Climate)) OR "Nature Based Solutions" OR "Nature Based Solution" OR "Nature-based Solution" OR "Nature-Based Solutions" OR NatureBasedSolutions OR NatureBasedSolution OR NatureBasedSols OR ("Nature Based" AND ("Climate Change" OR ClimateChange OR "Climate Policy" OR ClimatePolicy OR ClimateAction OR "Climate Action" OR ActonClimate)) OR #NCS) AND NOT (job OR jobs OR Photography OR Art OR Photooftheday OR photographer OR Futureofwork OR employee OR employment)

Climate Change mentions were triggered by the following boolean string: ((climate AND (change OR changing OR fluctuate OR fluctuating OR Policy)) OR climatechange OR "global warming" OR globalwarming OR climateaction OR actonclimate OR "ice cap" OR "ice caps" OR IPCC OR "1.5 Degree" OR "1.5 degrees" OR "Paris Agreement" OR ParisAgreement OR COP24 OR COP23 OR climaterisk OR "greenhouse gas" OR "greenhouse gases" OR greenhousegas OR greenhousegases OR emission OR emissions OR climatepolicy)

Supplementary Note 1

NCS activities are categorized into pathways: discrete and quantifiable bundles of activities that avoid greenhouse gas (GHG) emissions and/or increase carbon sequestration.[116]

All NCS Pathways are categorized by biomes:

- **Wetlands** include freshwater ecosystems, such as peatlands and freshwater mineral wetlands, as well as estuarine and coastal marine ecosystems, such as mangroves, tidal marshes, and seagrass meadows. (Note that forest, grasslands, and savanna wetlands and croplands farmed under saturated conditions are considered wetlands.)
- **Croplands** include any lands currently managed for annual or perennial crops, including agroforestry systems such as cacao, coffee, and intercropping.
- **Grasslands, Shrublands, and Savannas** include prairies, steppes, shrublands, tundra, savannas, and other habitats with tree cover < 25%. This includes grazed and ungrazed lands.
- **Forests** include any land that is not considered cropland with tree cover naturally > 25%, including tropical rainforests, dry forests, boreal forests, and tree plantations managed for wood products.
- **Oceans** include the marine biota and sediments in the open ocean.

All NCS Pathways are also categorized by conservation action [116,117]

- **Protect** "refers to pathways that prevent the loss of native ecosystems."
- **Manage** "refers to pathways that avoid GHG emissions or enhance carbon sinks on working lands and waters through improved management practices."
- **Restore** "refers to pathways that expand the spatial extent of native cover types, including forest and non-forest ecosystems, to areas from where they had previously been lost as a result of human activity."

A full list of NCS pathways and activities by biome and conservation action can be found here.

Supplementary Note 2

Below, NCS principles are applied to the Family Forest Carbon Program (FFCP), an exemplar NCS program launched by the American Forest Foundation (AFF) and The Nature Conservancy (TNC), and monitored through the Voluntary Carbon Standard (VCS).

Principle 1: NCS are Nature-based

Principle 1.2: NCS result from the **human stewardship of ecosystems**.

FFCP works directly with landowners and natural resource professionals to design and implement sustainable forest management plans on privately owned working forests. This is demonstrated in the periodic harvests shown in Figure SM3.

Principle 1.2: NCS do **not move ecosystems further from their natural state**.

All FFCP practices are designed in consultation with leading forest management professionals to maintain the critical functions of working forests, including a suite of ecosystem services. FFCP practices specifically do not allow for conversion away from forested land use or establishment/enrollment of plantations.

Principle 2: NCS are Sustainable

Principle 2.1: NCS **sustain biodiversity**.

FFCP practices are designed with attention to non-timber outcomes of forest management, including provision of high-quality wildlife habitat. Management of individual enrolled properties is governed by a sustainable forest management plan, which assesses and plans for biodiversity conservation at the forest-stand scale.

Principle 2.2: NCS **sustain food production**.

FFCP strategically minimizes impact on sustainable food production in two ways. First, projects are only applicable on land already in a forested land use. Second, in piloting reforestation and agroforestry practices, FFCP limits enrollment to agricultural land designated as marginal, to avoid conversion of high-quality agricultural lands.

Principle 2.3: NCS **sustain fiber and wood production**.

FFCP practices are specifically designed to maintain and enhance the productivity of working forests. As a result, practices are expected to increase forest product yields in the long term, as demonstrated by timber yields and carbon stocks in enrolled properties shown in Figure SM3

Principle 2.4: NCS **sustain climate adaptation** services**.**
FFCP practices are written to enhance and accentuate the species and structural complexity of enrolled forests, as documented in the sustainable forest management plans drafted for all enrolled properties. Increasing forest complexity has long been recognized as a way to increase forest resiliency and allow for a great breadth of future adaptation.

Principle 3: NCS are Climate-additional

Principle 3.1: NCS **provide additional climate mitigation that would not happen without human intervention**.
The methodology FFCP uses to quantify carbon emissions reductions[1] ensures additionality by comparing enrolled forests to non-enrolled forests with similar starting conditions, as shown in Figure SM3. Emissions reductions are calculated from ex-post measurements of forest carbon stock change on both FFCP-enrolled and non-enrolled properties, to show the direct result of enrollment in the program.

Principle 3.2: NCS **provide durable mitigation.**
FFCP uses several strategies to ensure that the mitigation delivered is durable. First, the program adheres to the VCS 100-year permanence requirement (VCS Non-Permanence Risk Tool [2], section 2.1.1). Second, the program invests in long-term engagement and education of landowners regarding principles of sustainable forest management, continuing after the completion of landowner's contracts and through ownership changes. Finally, the program is exploring options for compensating any reversals experienced post-contract, e.g., horizontal stacking of emissions reductions.

Principle 3.3: NCS **are not used to compensate for readily abatable emissions**.
FFCP conducts a rigorous vetting process for potential carbon credit buyers, to ensure they are following the established NCS hierarchy[3]. Recognizing their own limitations, AFF and TNC are also deeply engaged with demand-side initiatives like VCMI[4] and others, which provide universal, standardized best practices for sales of carbon credits.

Principle 4: NCS are Measurable

Principle 4.1: NCS are quantified **in terms of cumulative effects on radiative forcing**.
VM0045 was subject to a thorough and rigorous peer-review process, which sought to ensure that all relevant emissions are considered, that accounting principles are sound and accurate, and that estimated emissions reductions represent a realistic assessment of all elements of radiative forcing.

Principle 4.2: NCS **accounting is conservative**.
VM0045 has been reviewed and approved by Verra, which explicitly requires methodologies to adhere to the guiding principle of conservativeness in all calculations (VCS Standard[5], section 2.2).

Principle 4.3: NCS with **uncertainty ranges greater than the estimated climate mitigation** should be flagged as emerging.

FFCP explores many potential practices, but only implements those where estimated emissions reductions are far larger than the uncertainty associated with those estimates.

Principle 4.4: NCS accounting **avoids double-counting**.
VM0045 has been reviewed and approved by Verra, which explicitly prohibits overlapping NCS/ACOLU activities (VCS Standard[5], section 3.11.3).

Principle 5: NCS are Equitable
Principle 5.1: NCS **respect human rights**.
FFCP is implementing a strategic Diversity, Equity, Inclusion, and Justice plan, with the intention of engaging a wide range of landowners equitably. Furthermore, the program facilitates relationships between landowners and foresters, who have a fiduciary relationship with the landowner, thereby supporting informed decision-making by landowners, interested parties, and rights-holders with a spectrum of technical forestry experience.

Principle 5.2: NCS **respect Indigenous self-determination**.
FFCP is currently working with the National Indian Carbon Coalition (Foundation 2017) to explore how the FFCP model could offer access to carbon financing while ensuring Indigenous self-determination.

Supplementary References

1. Shoch, D. *et al*. *VM0045 Methodology for Improved Forest Management through Reduced Impact Logging v1.0 - Verra*. https://verra.org/methodology/vm0035-methodology-for-improved-forest-management-through-reduced-impact-logging-v1-0/ (2022).

2. Verified Carbon Standard. *AFOLU Non-Permanence Risk Tool*. https://verra.org/wp-content/uploads/2019/09/AFOLU_Non-Permanence_Risk-Tool_v4.0.pdf (2019).

3. Cook-Patton, S. C. *et al.* Protect, manage and then restore lands for climate mitigation. *Nat Clim Change* **11**, 1027–1034 (2021).

4. Voluntary Carbon Markets Integrity Initiative. *VCMI Provisional Code of Practice: Draft For Public Consultation and Corporate Road Testing*. https://vcmintegrity.org/wp-content/uploads/2022/06/VCMI-Provisional-Claims-Code-of-Practice.pdf (2022).

5. Verified Carbon Standard. *VCS Standard*. https://verra.org/wp-content/uploads/2022/12/VCS-Standard-v4.4-FINAL.pdf (2023).
